# Parenting Programs for the Prevention of Child Physical Abuse Recurrence: A Systematic Review and Meta-Analysis

**DOI:** 10.1007/s10567-017-0232-7

**Published:** 2017-04-04

**Authors:** Kristina Vlahovicova, G. J. Melendez-Torres, Patty Leijten, Wendy Knerr, Frances Gardner

**Affiliations:** 1 University of Oxford, Oxford, UK; 2 University of Warwick, Coventry, UK; 3 UvA University of Amsterdam, Amsterdam, Netherlands

**Keywords:** Child physical abuse, Parenting program, Systematic review, Meta-analysis

## Abstract

Child physical abuse is an issue of global concern. Conservative estimates set global prevalence of this type of maltreatment at 25%, its consequences and cost to society escalating with increasing frequency and severity of episodes. Syntheses of the evidence on parenting programs for reducing rates of physical abuse recidivism have, to date, not been able to establish effectiveness. Paucity of data and inconsistent inclusion criteria in past reviews made meta-analysis often impossible or uninformative. The current systematic review updates prior reviews and overcomes some of the methodological issues they encountered by pooling trial-level data from a well-defined scope of trials of parenting interventions aimed at preventing the re-abuse of children by parents with substantiated or suspected physical abuse history. Randomized controlled trials and rigorous non-randomized designs were sought via nine online databases, two trial registries, several clearinghouses and contact with experts. A total of fourteen studies of variable quality were included in this review, four of which had outcomes that enabled meta-analysis. Overall, this review presents evidence supporting the effectiveness of parenting behavioral programs based on social learning theory for reducing hard markers of child physical abuse recidivism. Meta-analysis found that the absolute risk reduction in risk of recidivism was 11 percentage points less for maltreating parents who undergo parenting programs (RD = −0.11, 95% CI [−0.22, −0.004], *p* = 0.043, *I* ^2^ = 28.9%). However, the pooled effect size was not statistically significant when calculated as a risk ratio (0.76, 95% CI [0.54, 1.07], *I* ^2^ = 38.4%). Policy makers and practitioners should be made aware that this intervention method is backed by promising evidence featuring modest yet significant reductions in hard markers of child physical abuse, even though the methodological robustness of these findings should be further explored in future research.

## Introduction

Child physical abuse is defined as the intentional use of physical force against a child, including hitting, beating, kicking, shaking, biting, scalding, burning, poisoning, and suffocating, often performed under the guise of discipline or punishment (WHO 2006). Worldwide cross-sectional surveys estimate that nearly one in four adults report experiencing physical abuse as children (WHO 2014; Butchart and Mikton 2014). Recent data from Egypt, India and the Philippines indicate that, in these countries, 26, 36 and 21% of parents, respectively, report hitting children with an object as a form of punishment (WHO 2014). Estimates of violence against children, which includes moderate to severe physical abuse, find that a minimum of 64% of 2–17-year-old children in Asia, 56% in Northern America, 50% in Africa, 34% in Latin America, and 12% in Europe experienced some form of violence in the last year (Hillis et al. 2016). These prevalence rates are not only high—they are also likely to be underestimates, as measurement errors, stigma and social normativity tend to mask the true magnitude of the problem (Finkelhor et al. 2014; Townsend and Rheingold 2013; Cicchetti and Toth 2005). Physical violence in particular is rarely reported and largely hidden: prevalence of physical abuse is over 75 times higher when assessed with victims’ self-reports rather than official reports (Stoltenborgh et al. 2013); and only the most severe cases tend to come to the attention of Child Protection authorities, if such authorities exist in the community at all.

The societal burden of child physical abuse is exorbitant—the lifetime economic cost for all new cases of abuse in one calendar year in the US has been estimated at $124 billion (Fang et al. 2012). Global estimates of the cost of this type of abuse in particular are not yet available, but a recent economic evaluation of the damage of violence against children (combining physical, psychological and sexual abuse only) has set the figure at $7 trillion, or up to 8% of global GDP (Pereznieto et al. 2014). The consequences of child physical abuse are costly, numerous, and severe—physical injury, disability, poor cognitive and socio-emotional outcomes, behavioral and mental health problems throughout the lifespan, perpetuation of abuse cycles, and even death are linked to having experienced abuse as a child (Gilbert et al. 2009; Gershoff 2010; Holmes et al. 2005; Repetti et al. 2002; Runyon et al. 2004; UNICEF 2006). While milder forms of physical abuse might have impairing consequences, there is an established dose–response relationship between experience of physical abuse in childhood and poor outcomes—that is, the most severe and persistent experiences of physical abuse are associated with the poorest outcomes (Norman et al. 2012). It is also known that violence breeds more violence, even across generations—children who have experienced physical abuse are most at risk of re-experiencing it (Hindley et al. 2006); and parents with a history of abuse during childhood are twice as likely to be reported to CPS for child maltreatment (Widom et al. 2015). It is therefore of vital importance to find effective interventions to prevent the recurrence of child physical abuse and break this cycle of violence.

Parenting programs are one such intervention. They are aimed at improving the quality of the parent–child relationship and preventing re-abuse by changing parenting attitudes, practices, and skills, as well as reducing parent–child conflict, coerciveness and parenting stress, improving parental psychosocial functioning, improving family dynamics and reducing child behavior problems (Barlow et al. 2006a; Montgomery et al. 2009). These interventions are generally based on Attachment Theory (Bowlby 1969), Learning Theory (Skinner 1950), and/or Social Learning Theory principles (Bandura 1971), though the latter informs most parenting interventions aimed to reduce child abuse. Most central in this is Patterson’s (1982) coercion hypothesis, which states that abuse might result from a repeating pattern of coercive parent–child interactions in which both the parent and the child escalate their violent behavior (Brinkmeyer and Eyberg 2003). On the side of the parent, the escalating coercive behavior springs from a belief that their child is defiant and unresponsive to less harsh forms of discipline. As children comply, parents may incorrectly believe that this strategy—and no other—works, and they therefore continue to use it (Crouch and Behl 2001). Parenting programs intend to break this cycle by promoting parental sensitivity, modifying parental attitudes, changing parental attributions, teaching adequate disciplining techniques, and increasing the use of positive parenting skills.

Prior reviews have found parenting programs to be promising strategies for reducing recurrence of child physical abuse. Four reviews in particular inspire the current review and meta-analysis. Barlow et al. (2006b) conducted a high-quality systematic review of individual- and group-based parenting programs to prevent child physical abuse and neglect recidivism, and reduce risk factors associated with re-abuse. This synthesis of RCTs revealed that, overall, parenting programs are a promising treatment strategy for preventing new incidents of abuse in families with a history of physical abuse—but not neglect. Furthermore, parenting programs were found effective in reducing risk factors associated with re-abuse when delivered to families with suspected or substantiated history of abuse. However, because their search resulted in a highly heterogeneous and limited set of trials, authors elected against conducting a meta-analysis of outcomes.

Another systematic review focusing specifically on corporal punishment (i.e., physical pain applied to correct or punish a child’s behavior) was conducted in Brazil (Santini and Williams 2016). It found 18 studies using different methodologies to evaluate the effectiveness of parenting programs to reduce corporal punishment, with all studies reporting medium to large reductions (*d* = .54–2.17). Nevertheless, the authors of this review also opted against conducting a meta-analysis at the time due to insufficient trial-level data reported by the included studies.

New evidence from the last decade has provided additional trials with enough clinical homogeneity to justify meta-analysis. Chen and Chan (2015) conducted an updated review of parenting programs for the treatment of child abuse, and attempted a meta-analysis of abuse recurrence outcomes (among others), finding that parenting programs successfully reduced substantiated and self-reported child maltreatment reports *(d* = .208). Parenting programs were also found to reduce risk factors—specifically ineffective parenting—and enhance protective factors such as endorsement of appropriate child-rearing attitudes, positive parenting, and parent–child interaction. However, given the clinical diversity of the interventions modalities included in their models, their meta-analyses also exhibited high degrees of statistical heterogeneity (*I* ^2^ = 75.6; *p* < .001), suggesting a need for a more tailored approach to understanding intervention modalities.

A systematic review and meta-analysis published in 2015 (Euser et al. 2015) identified 23 RCTs that tested the effect of 20 different programs (including but not limited to parenting programs) on child maltreatment prevention and/or reduction (including but not limited to physical abuse). It found a small but significant effect in favor of treatment (*d* = .13, 95% CI [0.05, 0.21]), but again, statistical heterogeneity was too high to indicate the true effect of these programs (*Q* = 56.06, *p* < .01). Additionally, trim-and-fill analysis of publication bias found that, after adjusting the results of 9 studies with small sample sizes, the pooled effect was greatly diminished (*d* = 0.02, 95% CI [−0.06, 0.11]), suggesting publication bias favoring the publication of smaller studies with significant findings.

Prior reviews suggest the potential effectiveness of indicated parenting programs to prevent child physical re-abuse. However, the body of evidence to date has not been large enough or evaluated with sufficient rigor to corroborate these findings. Additionally, the only meta-analyses that have been conducted (i.e., Chen and Chan 2015; Euser et al. 2015) suffered from problems resulting from a scope too wide and a level of heterogeneity too high to produce results with substantive value. The importance of conducting this review thus springs from two necessities: (1) to provide an up-to-date synthesis of the research on child physical re-abuse prevention using parenting programs, and (2) to overcome the methodological limitations that prior reviews have encountered by narrowing the scope of this review only to those interventions strictly based on SLT to enable combination of trial outcomes into a meta-analysis with less heterogeneity, thus producing a valuable reading of the cumulative evidence. The value of meta-analysis lies in its ability to estimate a mean effect of the interventions, thus providing a helpful basis by which to understand how effective programs could be when implemented in practice settings, and the degree to which new programs offer a meaningful advantage over existing interventions. Moreover, many reviews of complex interventions—such as parenting programs to reduce re-abuse—focus on a diversity of programs united by a similar theory of change (Bonell et al. 2016). This is an analytically helpful approach as it focuses on testing the underlying principles, which are thought to make interventions effective. In this study, we provide a broad test as to whether a theory of change, when implemented in the form of parenting interventions, has the potential to reduce recurrence of child maltreatment. Particularly, we focus on behavioral parenting programs (as opposed to non-behavioral programs, which might focus on transforming attitudes and attributions) to be better able to ascertain the effect of programs in this particular intervention modality without injecting problematic clinical heterogeneity in our collection of trials.

## Methods

### Criteria for Trial Inclusion and Exclusion

RCTs and quasi-experiments featuring a high-quality statistical matching technique to simulate randomization (e.g., Propensity Score Matched designs) were acceptable for inclusion in this review. Participants had to be parents (i.e., mothers, fathers, or other primary caregivers) of children aged 0–18, who have a suspected or substantiated report of child physical abuse. Both suspected and substantiated reports were acceptable for inclusion, as there is little difference between these groups in regards to their risk of recidivism (Drake et al. 2003; Kohl et al. 2009). Maltreatment history had to be supported by either (a) a police report, child protection referral, or other official agency report, (b) the self-report of an abusive parent or abused child, or (c) an above-threshold score in standardized instruments used for detection of child physical abuse, such as the Parent–Child Conflict Tactics Scale (CTS), the Child Maltreatment Interview Schedule (CMIS, one item on physical abuse), the ISPCAN Child Abuse Screening Tool (I-CAST), and the Alabama Parenting Questionnaire (APQ, corporal punishment, physical punishment, and minor/severe assault subscales). To ensure sufficient homogeneity to enhance comparability, behavioral parenting programs mostly based on SLT were selected for inclusion. Active and passive control conditions were acceptable—i.e., placebo, treatment as usual, alternative treatment, and wait-list controls.

As for outcomes, we focused particularly on physical abuse minding that different forms of abuse tend to co-occur (Jones et al. 2008; Oates and Bross 1995; Manly 2005). Samples showing multiple forms of abuse were included, provided that there was physical abuse present or suspected in at least 15% of the sample. This is an arbitrary threshold set by Oates and Bross (1995) and adopted by this review to maintain consistency with earlier literature, as well as to maximize the amount of studies that meet inclusion criteria. In cases where the amount of participants in the sample suspected of or reported for physical abuse was not stated, first authors were contacted to request more information. If the communication was unsuccessful, the trials were excluded, to err on the side of conservatism.

The primary outcome sought in this review were reports of child physical abuse recidivism, including re-report with police or child welfare/protection agencies, and/or self-report by parent or child. When official reports of recidivism were available (as opposed to self-reports by parents or children), they were prioritized. This is because official reports tend to be complete sets of data, available for all participants regardless of treatment completion status or attrition.

Additionally, proxy measures of physical abuse recurrence were considered acceptable indicators of re-abuse in the absence of direct re-abuse reports. These secondary measures include harsh parenting, physical punishment, and above-threshold scores in the standardized measures of child physical abuse that validly and reliably identify physical abuse occurrence: the PCTS, CMIS, APQ, and ICAST.

Although this review is solely interested in re-abuse outcomes, trials that did not collect re-abuse outcomes but met all other inclusion criteria were included in the final set of studies. In accordance with the Cochrane Handbook (Sect. 14.2.3), the presence or absence of outcomes is not a sufficient criterion to exclude studies from a systematic review (Higgins and Green 2011). Thus, outcomes related to changes in parent and child variables that are not indicative of abuse recidivism (such as parenting stress, or child problem behavior) are included but not synthesized, as this is not the focus of this review.

### Search Methods for Identifying Trials

#### Electronic Searches

Nine databases were searched to identify published studies from inception to April 10, 2015: MEDLINE, PsycINFO, EMBASE, PubMed, Cochrane Central Library, Campbell Library, ERIC, Sociological Abstracts, Social Service Abstracts, and CINAHL. The search string initially designed and adapted for use in other databases was:

((exp child abuse/) OR ((exp physical abuse/) AND (baby OR babies OR child* OR toddler* OR minor* OR adolescen* OR teen*)) OR (exp child abuse reporting/) OR (exp child discipline/) OR ((exp protective services/) AND (baby OR babies OR child* OR toddler* OR minor* OR adolescen* OR teen*)) OR (abusive head trauma) OR ((physical*) AND (maltreat* OR abus* OR mistreat*) AND (baby OR babies OR infan* OR child* OR toddler* or adolescen* OR teen* OR minor*)) OR ((intent*AND injur*) AND (baby OR babies OR infan* OR child* OR toddler* or adolescen* OR teen* OR minor*)) OR (corporal punishment ADJ3 (baby OR babies OR infan* OR child* OR toddler* or adolescen* OR teen* OR minor*))) AND ((exp Parent Training/) OR ((exp child-rearing practices/OR exp parent child relations/OR exp parental role/) AND (program* OR train* OR educat* OR promot* OR intervent* OR group* OR skill* OR support*)) OR ((mother* OR father* OR famil* OR caregiver* OR parent*) ADJ3 (program* OR train* OR educat* OR promot* OR intervent* OR group* OR skill* OR support*)))

This search favored sensitivity to capture all relevant studies on child physical abuse, regardless of level of prevention. This is because, judging by prior reviews (e.g., Chen and Chan 2015), it is not uncommon for participants at different levels of risk to be combined in the same trials. No methodological filters were applied to ensure that records were not missed due to poor reporting.

#### Grey Literature Searches

Trials for inclusion were also searched in the following clearinghouse websites: Child Welfare Information Gateway, Center for the Study and Prevention of Violence, National Clearing House of Families and Youth, California Evidence-Based Clearing House for Child Welfare, Child Welfare League of America, ChildTrends, Children and Families Research Center, and the Violence Against Children: United Nations Secretary General’s Study. Dissertations were included as long as they were captured by the database searches, and full-text papers could be retrieved either online or by contacting the author. Furthermore, twelve of the authors of the final set of included trials were contacted to identify ongoing or unpublished trials, of which eight replied with clarifications.

### Data Collection and Analysis

#### Selection of Studies

The first and second authors independently conducted the selection of studies for inclusion in this review in three stages. An initial title scan was conducted. Subsequently, the abstracts of seemingly relevant titles were scanned to determine whether they met the inclusion criteria. Finally, full-text copies of papers that appeared to meet criteria were reviewed. Uncertainties related to the appropriateness of studies for inclusion were resolved in consultation with co-authors.

#### Assessment of Risk of Bias in Included Studies

Critical appraisal of included studies was conducted. An adapted version of the Cochrane Risk of Bias Tool (Higgins and Green 2011) was used to assess the methodological robustness of studies. All of the dimensions of trials assessed by this tool (random sequence generation, allocation concealment, blinding, and reporting) were ranked as either “high risk,” “low risk,” or “unclear.”

#### Measures of Treatment Effects

Outcome data were presented as Cohen’s *d* effect sizes (Cohen 1969), if enough data were provided by authors in trial reports (i.e., means and standard deviations for continuous data, or count of new incidents and sample sizes of groups for dichotomous data).

We recalculated effectiveness associated with dichotomous outcomes as risk differences (also known as absolute risk reduction). This had several benefits, most importantly that unlike odds ratios and risk ratios, risk differences may be more readily interpretable as the absolute change in risk of an outcome, which may be more relevant from a policy and practice perspective. When risk differences were combined in a meta-analysis, we sensitivity-tested our findings as risk ratios as well. We also converted risk differences into the “number needed to treat” by taking the inverse of the risk difference (i.e., 1/RD). The number needed to treat is the number of families who would need to receive the intervention in order to prevent one incident of re-abuse (Higgins and Green 2011).

#### Unit of Analysis Issues

Some trials had multiple relevant treatment arms (e.g., parent–child interaction therapy and enhanced parent–child interaction therapy), and others had multiple relevant outcomes (e.g., parent self-report of re-abuse and child self-report of re-abuse). When these trials were included in the meta-analysis, the treatment arms (or outcomes) were combined. That is, the participants of the relevant treatment arms were added together (as were their respective counts of recidivist participants), and the multiple outcomes were averaged to produce one single measure of outcome. This is a reasonable course of action whenever treatment arms are similar versions of the same intervention and both treatment arms observe effects in the same direction (Higgins and Green 2011, Sect. 16.5.4).

#### Dealing with Missing Data

Missing data and dropouts were assessed for each included study, and the review reports the number of participants who have been included in the final analysis as a proportion of all participants in each study.

#### Assessment of Heterogeneity

To expose statistical heterogeneity in the meta-analysis (Higgins and Green 2011) we calculated the *I* ^2^ index, which indicates the amount of variability in the intervention effects. An *I* ^2^ index larger than .5 (i.e., 50%) indicates that caution should be exerted in making substantive inferences about the results of the meta-analysis. Due to the small number of studies included, we did not conduct any subgroup analyses (i.e., exploration of the effect of participant characteristics, or other contextual factors).

#### Data Synthesis

Abuse recidivism data are typically presented in two ways: recurrence of physical abuse can be expressed either as event data (i.e., presence or absence of re-abuse in a given time period), as time-to-event data (i.e., time to re-abuse incident), or both. Risk differences relating to re-abuse event data (both official re-reports and self-reports of recidivism by parents/children) were synthesized statistically using a random-effects meta-analysis model to account for heterogeneity.

Time-to-event data were not included in the meta-analysis. Although, theoretically, event and time-to-event data could be combined, not enough information was provided in trial reports to combine them without making ad hoc assumptions. Additionally, time-to-event data could not be meta-analyzed separately, as it was presented inconsistently (e.g., as nonparametric tests, such as log rank, or as hazard ratios), without enough information to facilitate conversion to one common unit, as variance and error were often missing from reports. Individual participant data would be required to compute these missing factors.

Finally, all other outcomes (harsh parenting, physical punishment, and scores on standardized abuse detection measures) were reported as standard mean differences with 95% confidence intervals whenever means and standard deviations were reported in the included trials.

## Results

### Results of the Search

**Fig. 1:**
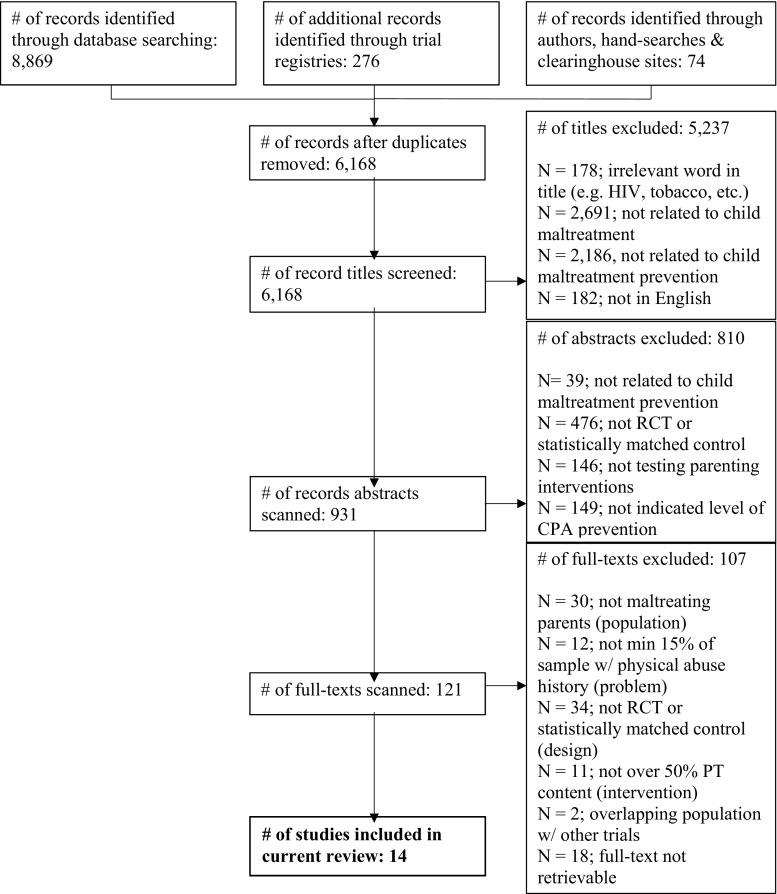
PRISMA flowchart of selection of studies for inclusion in systematic review

Online database searches yielded 8869 results. Searching trial registries and clearinghouse websites, contact with authors, and hand searching prior reviews added 424 hits. In total, after de-duplication, 6168 records were captured (see Fig. 1). The process of scanning records for eligibility was conducted in three stages by the first author and repeated independently by the second author. Initially, all titles were scanned and excluded if they held no direct relevance to this review (i.e., titles unrelated to child abuse, non-English language publications, and publications not concerned with child abuse treatment such as case studies, prevalence studies, risk factors analyses, descriptive studies, observational data reports, and evaluations of assessment tools). Then, abstracts of the remaining records were scanned and excluded if they (a) were not related to child abuse prevention, (b) were not RCTs or statistically controlled designs, (c) were not testing parenting programs, and (d) were not concerned with the indicated prevention of child physical abuse. In the third stage, full-text articles of the remaining 121 records were closely examined. An eligibility form was created to streamline and standardize this process. Inclusion ambiguities were resolved in collaboration with co-authors. After the sorting process was completed, 14 studies remained eligible and were included in this review (Table 1).

**Table 1:** Characteristics of included studies

First author (year)	Design	Intervention name	Comparison group	Child age	Dose	Setting	Re-abuse effect size
Brunk (1987)	RCT	Parent training	Multi-systemic therapy	–	6 weeks	Clinic	n/a
Chaffin (2004)	RCT (stratified)	PCIT and EPCIT	Standard community group	4–12	12–14 Sessions over 6 months	Clinic	RR = 0.57 [0.35, 0.95]
Chaffin (2011)	2 × 2 RCT	PCIT + SM	TAU	2.5–12	12–14 sessions over 6 months	Clinic	RR = 1.03 [0.69, 1.55]
Chaffin et al. (2012)	RCT	Safe care	Home visitation without SC components	0–12	Weekly for approx. 6 months	Center	HR = 0.74–0.83
Eagan (1983)	RCT	Child Management Program	TAU—case management	–	6 weeks	–	n/a
Hughes and Gottlieb (2004)	RCT	Incredible years	Wait-list control	3–8	8 Weekly 2-h sessions	Center	n/a
Jouriles (2010)	RCT	Project support	TAU	3–8	1.5 h Weekly for 8 months	Home	RR = 0.21 [0.03, 1.63]
Kolko (1996)	RCT	Individual child- and parent-CBT	Family therapy + community services	6–13	16 weeks	Clinic/Home	RR = 0.40 [0.17, 0.96]
MacMillan (2005)	RCT	Home visitation (nurses)	TAU	0–13	2 years	Home	RR = 0.77 [0.51, 1.14]
Mast (2014)	RCT	I-inTERACT	Internet resource comparison	3–9	Weekly for 6 months	Online	n/a
Runyon (2010)	RCT	Combined parent–child CBT	Parent-only CBT	7–13	16 weeks	Clinic	SMD = 0.01 [−0.50, 0.52]
Swenson (2010)	RCT	STEP-TEEN	Multi-systemic therapy	10–17	7 weeks	Center	RR = 2.10 [0.40, 10.84]
Terao (1999)	RCT	PCIT	Family preservation	–	12–14 Sessions over 6 months	Home	n/a
Wolfe (1981)	RCT	Child management program	Wait-list control	2–10	6 weeks	Clinic/Home	RR = 0.33 [0.02, 7.14]

(E)PCIT = (Enhanced) parent–child interaction therapy, SM = self-motivation, SC = safe care, CBT = cognitive behavioral therapy, TAU = treatment as usual, SMD = standard mean difference, RR = risk ratio, CI = confidence interval, APQ = Alabama parenting questionnaire, HR = hazard ratio

### Excluded Studies

The main reasons for exclusion were that (a) the trials did not use an RCT or statistically controlled design, (b) the population was not at least 15% physically abusive, (c) the intervention did not qualify as parent training or did not contain a majority of parenting content, and (d) the level of prevention was not indicated, but rather selective or universal (Table 2).

**Table 2:** Characteristics of excluded studies

First author, year	Reason for Exclusion
*Armstrong, 1999*	Not indicated level of prevention
*Barlow, 2007*	Not indicated level of prevention
*Barlow, 2013*	Not indicated level of prevention
*Barnes, 2013*	Not indicated level of prevention
*Barth, 2006*	Not >51% parenting intervention
*Bernard, 2010*	<15% sample physically abusive parents
*Bigelow, 2000*	Not RCT or statistically matched control
*Byrne, 2012*	Not RCT or statistically matched control
*Casanueva, 2008*	Not >51% parenting content in intervention
*Chaffin, 2001*	Not RCT or statistically matched control
*Chaffin, 2009*	Testing motivational component effect on retention
*Chaffin, * 2012b	Sample of this trial overlaps with Chaffin et al. 2012a
*Christoffersen, 2009*	Not RCT or statistically matched control
*Cicchetti, 2006*	Not indicated level of prevention; <15% physically abusive
*Constantino, 2001*	Not indicated level of prevention
*Dawe, 2007*	Not indicated level of prevention; <15% physically abusive
*Denicola, 1980*	Not RCT or statistically matched control
*Dubowitz, 2009*	Not >51% parenting content in intervention
*Duggan, 2004a*	Not indicated level of prevention
*Duggan, 2004b*	Not indicated level of prevention
*Duggan, 2007*	Not indicated level of prevention
*Eckenrode, 2000*	Not indicated level of prevention
*Edwards*-*Gaura, 2003*	Not indicated level of prevention
*Fantuzzo, 2007*	Not >51% parenting content in intervention
*Fergusson, 2005*	Not indicated level of prevention
*Fergusson, 2006*	Not indicated level of prevention
*Fergusson, 2013*	Not indicated level of prevention
*Fetsch, 1999*	Not RCT or statistically matched control
*Fraser, 2000*	Not indicated level of prevention
*Gavlick, 2003*	Not indicated level of prevention; <15% physically abusive
*Gershater*-*Molko, 2003*	Mixture of selective and indicated level of prevention
*Green, 2014*	Not indicated level of prevention
*Guterman, 2013*	Not indicated level of prevention
*Hakman, 2009*	Not RCT or statistically matched control
*Hall, 2004*	Not RCT or statistically matched control
*Harder, 2005*	Not RCT or statistically matched control
*Harnett, 2008*	Not RCT or statistically matched control
*Horton, 2013*	Not indicated level of prevention
*Horwitz, 2010*	Not RCT or statistically matched control
*Hughes, 2002*	Sample of this trial overlaps with Hughes and Gottlieb 2004
*Hulburt, 2013*	Inadequate control group (not maltreating population)
*Irueste*-*Montes, 1988*	Not RCT or statistically matched control
*Kim, 2008*	Not indicated level of prevention
*Knox, 2011*	Not indicated level of prevention
*Lanier, 2014*	Not RCT or statistically matched control
*Lau, 2011*	Not indicated level of prevention
*LeCroy, 2011*	Not indicated level of prevention
*Letarte, 2010*	Not indicated level of prevention; <15% physically abusive
*Linares, 2006*	Not RCT or statistically matched control
*Lind, 2014*	No report of % sample physically abused
*Lober, 1984*	Not RCT or statistically matched control
*Lowell, 2014*	Not indicated level of prevention
*Luthar, 2007*	Not indicated level of prevention
*Lutzker, 1987*	Not RCT or statistically matched control
*Maher, 2011*	Not RCT or statistically matched control
*Maher, 2012*	Not RCT or statistically matched control
*Meezan, 1998*	Not >51% parenting content in intervention
*Moss, 2011*	Intervention not aimed at modifying parenting abusive practices
*Nese, 2014*	Not RCT or statistically matched control
*Olds, 1997*	Not indicated level of prevention
*Polinsky, 2010*	Not RCT or statistically matched control
*Ramquist, 2010*	Not RCT or statistically matched control
*Reading, 2008*	Not RCT or statistically matched control
*Reynolds, 2003*	Inadequate control group (not maltreating population)
*Rivara, 1985*	Not RCT or statistically matched control
*Runyan, 2009*	Not indicated level of prevention
*Saldana, 2015*	<15% sample physically abusive parents
*Scott, 2012*	Not RCT or statistically matched control
*Self*-*Brown, 2012*	Not RCT or statistically matched control
*Shaeffer, 2013*	Not RCT or statistically matched control
*Smith, 1984*	Not RCT or statistically matched control
*Sprang, 2009*	Not indicated level of prevention
*Stronach, 2013a*	<15% sample physically abusive parents
*Stronach, 2013b*	No report of % sample physically abused
*Thomas, 2011*	No report of % sample physically abused
*Thomas, 2012*	Not RCT or statistically matched control
*Toth, 2002*	Intervention not aimed at modifying parenting abusive practices
*Timmer, 2005*	Not RCT or statistically matched control
*Timmer, 2006*	Not RCT or statistically matched control
*Walker, 2008*	Intervention does not qualify as parenting
*Wolfe, 1980*	Not RCT or statistically matched control

### Description of Included Studies

#### Study Designs

All 14 included studies were RCTs. One of the trials was a cluster-randomized trial (Chaffin et al. 2012, randomized at the Child Protection Service or CPS agency level), one was a 3-arm trial (Chaffin et al. 2004, comparing PCIT vs. enhanced PCIT vs. community standard), one was a 4-arm trial (Egan 1983, comparing parenting programs vs. stress management vs. parenting + stress management vs. wait-list control), and two were 2 × 2 stratified trials (Chaffin et al. 2011, randomized first to orientation group type and then to intervention type; Chaffin et al. 2012, randomized first to intervention type and then to coached vs. un-coached implementation).

#### Populations

All studies comprised a minimum of 15% physically abusive parents, bar one (Chaffin et al. 2012), which included mostly neglecting families and only 14% physically abusive families, but was still included in the review as it only missed this criterion by 1% and met all other inclusion criteria. Seven trials included exclusively physically abusive parents, and others ranged between 23% and 63%. The number of participants in each study ranged substantially, from 26 to 2176.

#### Interventions

The 14 trials evaluated 8 different SLT-based behavioral parent training programs. The content of the programs was reasonably similar, with a shared focus on teaching and practicing parenting skills and child management strategies to break cycles of coerciveness in parent–child interaction, although some programs also included modules on child health and safety practices (e.g., Chaffin et al. 2012). While most programs ran weekly sessions with a similar duration (between 1 and 2 h per session), the total duration of each program varied greatly, with 6 of the programs running for 4–8 months (Chaffin et al. 2004, 2011, 2012; Terao 1999; Jouriles et al. 2010; Mast et al. 2014; Kolko 1996; Runyon et al. 2010), some running for only 8 weeks (Hughes and Gottlieb 2004; Swenson et al. 2010; Egan 1983; Brunk et al. 1987; Wolfe et al. 1981), and one for over 2 years (MacMillan et al. 2005). Programs were delivered either individually, to groups, or both; and fully or partially delivered in the home (Jouriles et al. 2010; Kolko 1996; MacMillan et al. 2005; Wolfe et al. 1981; Terao 1999), healthcare or other clinics (Chaffin et al. 2004, 2011; Brunk et al. 1987, Kolko 1996, Wolfe et al. 1981, Runyon et al. 2010), community centers (Chaffin et al. 2012, Hughes and Gottlieb 2004, Swenson et al. 2010), and online (Mast et al. 2014). One trial did not report delivery setting (Egan 1983). The size of the samples also varied tremendously, with the smallest trial including only 26 participants (Hughes and Gottlieb 2004) while the largest included data on almost 2200 families (Chaffin et al. 2012).

#### Comparison Groups

Three trials used wait-list control groups (Egan 1983, Hughes and Gottlieb 2004; Wolfe et al. 1981). The wait period varied across trials: Egan (1983) had a 6-week waitlist, Wolfe et al. (1981) was 8 weeks, and Hughes and Gottlieb (2004) only offered treatment after 4 months. All of three trials offered usual agency services during the wait period. Five trials used “service-as-usual” or “treatment-as-usual” controls (Chaffin et al. 2004, 2011, 2012; Jouriles et al. 2010; MacMillan et al. 2005). Yet, what was offered as usual treatment differed greatly between trials, some of which would be best classified as alternative treatments. In the Chaffin et al. (2004, 2011) trials, “service-as-usual” controls were offered a non-SLT parenting group program. In Chaffin et al. (2012), “service-as-usual” included a behavioral skills training that resembled the treatment intervention in content, but not in structure, dose, or delivery format. In Jouriles et al. (2010), the control conditions varied from nothing to a parenting program alternative treatment. MacMillan et al. (2005) considered “treatment-as-usual” providing control parents with child physical abuse caseworkers, assessment of recidivism risk, education about parenting, and referrals to other services. The Terao (1999) trial offered a “family preservation group” alternative, comprising a range of services that did not include parent training.

Alternative treatments that were used as control groups included family therapy (FT, Kolko 1996), multi-systemic therapy (MST, Brunk et al. 1987; Swenson et al. 2010), Internet resources (IRC, Mast et al. 2014); none of which are primarily based on SLT or focus mainly on parenting training instruction. Only CPC–CBT (i.e., Combined Parent Child Cognitive Behavioral Therapy, the comparison treatment in Runyon et al. 2010) is based on the same theory as the interventions. Yet it was considered a valid alternative, since, reportedly, the amount of parent training was small compared to the parent-only version of the treatment (P-CBT).

#### Risk of Bias in Included Studies

The summary chart (Fig. 3) gives an overview of the quality of the evidence included in this review. Notably, three trials reported unsuccessful randomization (Chaffin et al. 2011; Brunk et al. 1987; Runyon et al. 2010); none of the trials bar one (MacMillan et al. 2005) detailed the method used for allocation concealment; trial attrition was between 2 and 23%; only four trials reported intention-to-treat analysis (Chaffin et al. 2012; Kolko 1996; MacMillan et al. 2005; Swenson et al. 2010); and none of the trials blinded participants or research personnel to treatment assignment, although blinding is virtually impossible to achieve in a trials of a psychosocial intervention. Lastly, five trials had small sample sizes and limited power to detect effects (Jouriles et al. 2010; Kolko 1996; Mast et al. 2014; Runyon et al. 2010; Wolfe et al. 1981).

### Effects of Interventions

#### Primary Review Outcomes: Re-abuse

Seven trials collected event data of official re-reports to CPS or similar agencies (Chaffin et al. 2004, 2011, 2012; Jouriles et al. 2010; MacMillan et al. 2005; Swenson et al. 2010; Wolfe et al. 1981). Parent and child self-reports of number of new abuse incidents were collected from one trial only (Kolko 1996), in which parents and children separately ranked from 1 to 4 the severity and frequency of the use of force and infliction of injury in the early and late stages of the intervention. The authors of the trial dichotomized these answers to presence or absence of at least one incident of abuse during the course of the intervention.

#### Meta-Analysis: Risk of Re-abuse in Active Versus Treatment as Usual Trials

**Fig. 2:**
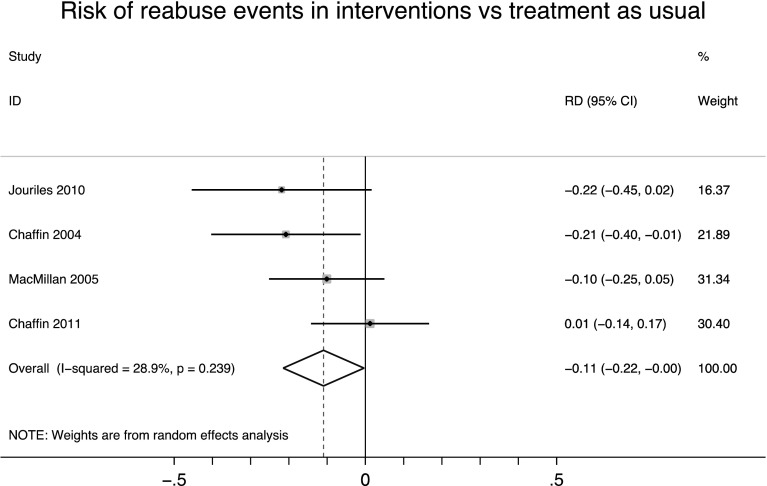
Risk of re-abuse events in parenting programs versus treatment as usual Chaffin et al. (2004) combined EPCIT and PCIT conditions

Of these seven trials, we meta-analyzed risk differences for four trials comparing manualized interventions against treatment as usual, and measuring outcomes via re-reports or referrals to CPS (Jouriles et al. 2010; Chaffin et al. 2004, 2011; MacMillan et al. 2005; see Fig. 2). On the whole, the absolute reduction in risk of recidivism was 11 percentage points less and was statistically significant (RD = −0.11, *p* = 0.043, 95% CI [−0.22, −0.004]). Another way of understanding these results is that about nine families would need to be treated to prevent one incident of re-abuse. Heterogeneity was notable, but not necessarily large (*I* ^2^ = 28.9%). When we conducted sensitivity analyses as risk ratios, findings were no longer significant (RR = 0.76, 95% [CI 0.54, 1.07], *I* ^2^ = 38.4%).

#### Narrative Synthesis: Risk of Re-abuse in Active Versus Active Trials

An additional three trials (Kolko 1996; Wolfe et al. 1981; Swenson et al. 2010) compared included parenting interventions against another active intervention, but we did not meta-analyze these as the comparator would have been too clinically heterogeneous to be interpretable, and each of the three trials measured re-abuse in a different way. When the relevant, SLT-oriented parenting programs were compared against the other active treatment arms (e.g., family preservation groups, family therapy, multi-systemic therapy), effects were inconsistent. Two studies yielded non-significant risk differences: Wolfe et al. (1981) (RD = −0.125, 95% CI [−0.411, 0.161]) and Swenson et al. (2010) (RD = 0.050, CI [−0.058, 0.158]), whereas Kolko (1996) showed a significant positive effect when compared against specific family therapy (RD = −0.350, CI [−0.647, −0.054]).

#### Narrative Synthesis: Time to Re-abuse Recidivism

Three trials provided data on the amount of time before a new recidivism episode (time-to-event data). Chaffin et al. (2004) found that PCIT significantly delayed re-abuse when compared to the standard community group condition (log rank = 6.2, *p* = 0.02; unit = days). Furthermore, although PCIT delayed time to re-abuse better than the Enhanced PCIT condition in which ancillary services were also offered, the comparison between EPCIT and the community group condition did not approach significance (log rank = 2.3, *p* = 0.13).

In a different study of PCIT, Chaffin et al. (2011) found longer survival for the PCIT with self-motivation orientation group relative to PCIT without self-motivation (hazard ratio = 0.11, *p* < .05; unit = days), to service-as-usual with self-motivation (hazard ratio = 0.10, *p* < .05), and to service-as-usual without self-motivation (HR = 0.20).

**Fig. 3:**
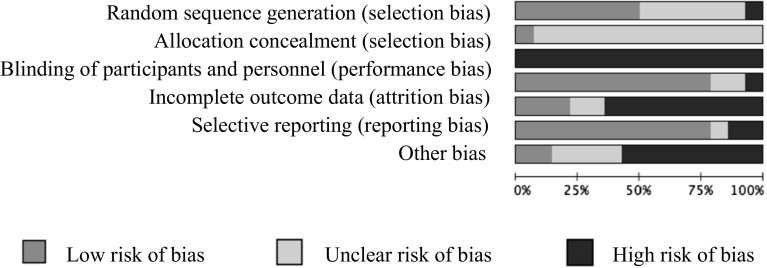
Risk of bias graph: summary of authors’ rankings of included trials on different dimensions of risk of bias, presented as percentages across all included studies

Lastly, results from Chaffin et al. (2012) showed a longer time to re-abuse for the intervention (SafeCare) over a 6-year follow-up period when compared to a different home-visitation intervention (hazard ratio = 0.74–0.83). Coaching did not make a significant difference to these effects (Fig. 3).

#### Secondary Review Outcomes: Harsh Parenting and Physical Punishment

Runyon et al. (2010) collected scores on the APQ (corporal punishment subscale) but did not find a significant difference between P-CBT and CPC–CBT in terms of corporal punishment (*N*_int_ = 26, *M*_int-post_ = 4.47, SD = 2.07 vs. *N*_ctrl_ = 34, *M*_ctrl-post_ = 4.44, SD = 2.1; *d* = 0.01, 95% CI [−0.50 to 0.52]). Since the confidence interval crosses the point of no effect (i.e., 0), these results are statistically non-significant.

Swenson et al. (2010) collected scores for the physical aggression, minor assault, and severe assault subscales of the CTS. Authors did not provide means and standard deviations, but they reported the significance level of between-group differences and the standardized mean difference (SMD), expressed as Cohen’s *d*. Physical aggression (as reported by youth) differed significantly between STEP-TEEN and MST groups, favoring MST (*p* < 0.01, *d* = 0.21). Minor assault (as reported by youth) also differed significantly between groups in favor of MST (*p* < 0.01, *d* = 0.14), as did severe assault (as reported by youth; *p* < 0.01, *d* = 0.54).

Jouriles et al. (2010) also collected CTS scores from the corporal punishment subscale. Results strongly favored Project Support versus service-as-usual at the post-intervention mark (*N*_int_ = 17, *M*_int-post_ = 0.87, SD = 0.93 vs. *N*_ctrl_ = 15, *M*_ctrl-post_ = 1.64, SD = 1.04; *p* < 0.05, *d* = 0.86, 95% CI [0.15–1.53]).

#### Secondary Review Outcomes: Other Parent-Related Outcomes

Other parent-related outcomes collected in the trials that were not indicative of recidivism were: child abuse potential (Chaffin et al. 2004; MacMillan et al. 2005; Terao 1999), child-rearing attitudes (MacMillan et al. 2005), hospitalizations related to maltreatment (MacMillan et al. 2005), out-of-home placements (Swenson et al. 2010), observation measures of negative and/or positive parenting behaviors (Chaffin et al. 2004, 2011; Egan 1983; Hughes and Gottlieb 2004; Jouriles et al. 2010; Mast et al. 2014), parent mental health (Egan 1983; Jouriles et al. 2010; Swenson et al. 2010), parent autonomy support (Hughes and Gottlieb 2004), family relations and functioning (Brunk et al. 1987; Egan 1983; Kolko 1996; MacMillan et al. 2005), parenting stress (Brunk et al. 1987), parent locus of control (Jouriles et al. 2010), parent anger (Kolko 1996), and social support (Brunk et al. 1987; MacMillan et al. 2005; Swenson et al. 2010). We did not synthesize these further as they are not directly predictive of re-abuse or abusive behaviors.

## Discussion

This review was conducted to strengthen our understanding of the effectiveness of SLT-based behavioral parenting programs for preventing child physical abuse recurrence. Methodologically, it overcomes several important challenges encountered in prior reviews (e.g., Barlow et al. 2006b; Chen and Chan 2015), by including evidence from the last decade, selecting trials for inclusion with stringent criteria, and conducting an informative meta-analysis featuring limited statistical and clinical heterogeneity. The results of this review suggest that behavioral parenting programs are modestly but significantly effective strategies for reducing hard markers of recidivism in physically abusive families. Our meta-analysis found recidivism to be 11% lower for CPS referred families who received SLT-based behavioral parenting training. While this figure is modest, it is important to recognize its magnitude given the complicated nature of child welfare systems and the multiple high risks to which referred families tend to be exposed to. Granted, more extensive and better-quality research is needed to understand the effectiveness of this intervention modality, and thus establish its effectiveness more robustly. While we were only able to include four studies in the meta-analysis, a better-powered analysis may also have been able to understand not only whether this intervention modality is effective, but also the differences between specific interventions that might make them more or less effective.

A few limitations of this review must be highlighted. First, the included trials were conducted exclusively in the US or Canada. This is not uncommon in the field of child maltreatment: in a systematic review of reviews by Mikton and Butchart (2009), it was established that 90% of trials of child maltreatment interventions were conducted in high-income countries. Orienting future systematic reviews to include trials in languages other than English might help ensure that research from other settings is captured, thus reducing the possibility that geographic homogeneity is an artifact of the search criteria. On the other hand, this review could serve as a starting point for a regional analysis of program effectiveness in this region. In that case, search criteria should be expanded to include child neglect, seeing as it is the mode reason for CPS reports in this region.

Second, only half of the included trials had a follow-up assessment, of which only 14% only followed participants for more than 6 months. Only one notably strong trial (Chaffin et al. 2012) had a longitudinal design, with a 6-year follow-up period. Longer follow-up periods in other similar trials would be necessary to understand the long-term effects of parenting programs.

Third, some decisions made during the selection of studies for inclusion might have introduced bias in this review. For instance, one of the included trials (Chaffin et al. 2012) barely met participant inclusion criteria—the proportion of physically abusive parents was 14% instead of the set minimum of 15%. However, given that only 1% was missing in this instance, an exception was made. Another exception was made for the MacMillan et al. (2005) trial, where the number of physically abusive parents was not reported, but the overall quality of the trial and perfect fit with other inclusion criteria prompted its exceptional inclusion. Future reviews should revisit the conceptual framework for setting the thresholds for inclusion at 15%, considering the low reporting rates for this specific type of abuse.

Lastly, while the statistical heterogeneity in the meta-analysis was low, the clinical heterogeneity present in the set of included studies might need to be carefully considered. The interventions grouped under the umbrella category “parenting programs” included a diversity of components, dosages, delivery settings, and other elements. Nonetheless, this variability is advantageous for the purposes of exploring the effectiveness of the theory of change (i.e., the underlying principles of SLT-based programs), as opposed to any one particular intervention modality or program. This said, when the evidence base is large enough, future reviews should include subgroup analysis so as to better understand how intervention and participant characteristics might be influencing the observed effect. For instance, it would be interesting to explore the differential effects that parent training might have on different types of families (e.g., families with substance abuse issues, single-parent families). Meta-analyses of pooled individual-level data from trials on parenting programs could elucidate differences between types of participants in subsequent synthesis efforts.

Future research should also focus on understanding how parenting programs work and how their effectiveness can be improved, by exploring the specific mechanisms through which programs reduce or prevent child maltreatment. This is because parenting interventions are complex intervention packages that include multiple interacting components related to parenting knowledge, principles, and skills (Kaehler et al. 2016). Knowing which core components are driving effectiveness can help optimize interventions by making them briefer, more effective and cost-effective, and improving implementation, reach, uptake, replicability, and sustainability of effects (Elliott and Mihalic 2004; Leijten et al. 2015, Glasziou et al. 2008; Linnan and Steckler 2002). Bentovim and Elliott (2014) initiated the important task of identifying core components of parenting interventions by employing a “distillation and matching” technique on a few selected RCTs that found parenting training effective for the treatment of physical abuse recidivism. Methodologies such as meta-analysis of components (e.g., Kaminski et al. 2008) could also be used in this context to systematically and retrospectively explore which intervention components are related to the strongest effect sizes.

This review ought to be replicated and updated as more and better-quality evidence becomes available. However, at present, it is defensible to conclude that targeting the parent–child relationship through SLT-based behavioral parenting programs can be an effective treatment for preventing recurrence of child physical abuse—at least in a North American context.
